# Comprehensive analysis of differential long non-coding RNA and messenger RNA expression in cholelithiasis using high-throughput sequencing and bioinformatics

**DOI:** 10.3389/fgene.2024.1375019

**Published:** 2024-05-14

**Authors:** Yanbo Sun, Conghui Xu, Jing Luo, Shumin Li, Shi Chen, Yunyun Cen, Pengyuan Xu

**Affiliations:** ^1^ Department of Gastrointestinal Surgery, The Second Affiliated Hospital of Kunming Medical University, Kunming, China; ^2^ School of Medicine, Yunnan University, Kunming, China; ^3^ Department of Gastrointestinal Surgery, Qujing No. 1 People’s Hospital, Qujing, Yunnan, China

**Keywords:** ceRNA, cis-and trans-regulation, etiology, gallstone, lncRNA

## Abstract

**Background:**

The etiology of gallstone disease (GSD) has not been fully elucidated. Consequently, the primary objective of this study was to scrutinize and provisionally authenticate the distinctive expression profiles of long non-coding RNAs (lncRNAs) and messenger RNAs (mRNAs) in GSD.

**Methods:**

RiboNucleic Acid (RNA) sequencing was used on four paired human gallbladder samples for the purpose of this study. Differentially expressed lncRNAs (DElncRNAs) and mRNAs (DEmRNAs) were identified and subjected to analysis of their biological functions. The Pearson’s correlation coefficients between DElncRNAs and DEmRNAs were computed to construct a co-expression network delineating their associations. Furthermore, both cis- and trans-regulatory networks of selected lncRNAs were established and visualized. Additionally, a competing endogenous RNA (ceRNA) regulatory network was constructed. To validate the RNA-sequencing data, we performed a Quantitative Real-time Polymerase Chain Reaction (RT-qPCR) on 10 paired human gallbladder samples, assessing the expressions of the top 4 DEmRNAs and DElncRNAs in gallstone and control samples.

**Results:**

A total of 934 DEmRNAs and 304DElncRNAs were successfully identified. Functional enrichment analysis indicated a predominant involvement in metabolic-related biological functions. Correlation analysis revealed a strong association between the expressions of 597 DEmRNAs and 194 DElncRNAs. Subsequently, both a cis-lncRNA-mRNA and a trans-lncRNA-Transcription Factor (TF)-mRNA regulatory network were meticulously constructed. Additionally, a ceRNA network, comprising of 24 DElncRNAs, 201 DEmRNAs, and 120 predicted miRNAs, was established. Furthermore, using RT-qPCR, we observed significant upregulation of AC004692.4, HECW1-IT1, SFRP4, and COMP, while LINC01564, SLC26A3, RP1-27K12.2, and GSTA2 exhibited marked downregulation in gallstone samples. Importantly, these findings were consistent with the sequencing.

**Conclusion:**

We conducted a screening process to identify DElncRNAs and DEmRNAs in GSD. This approach contributes to a deeper understanding of the genetic factors involved in the etiology of gallstones.

## 1 Introduction

Gallstone disease (GSD) is a common global ailment, with incidence rates reaching 10%–15% in developed countries and varying between 4% and 13% in different regions of China (Portincasa et al., 2006; Sun et al., 2009; Chen et al., 2012; Stinton and Shaffer, 2012; Xu et al., 2012; Zhu et al., 2014) Epidemiological investigations have established that GSD arises from a combination of genetic, environmental, dietary, and other factors (Portincasa et al., 2006; Zhu et al., 2014; Di Ciaula et al., 2018). Although extensive research has been undertaken to elucidate its mechanisms, encompassing disorders such as cholesterol-bile acid imbalance, structural disruptions or functional impairments in the human intestine-liver axis, aberrant gallbladder contraction function, perturbations in microbiota, metabolic imbalances, and the influence of estrogen, the precise pathogenesis is still not fully understood (Portincasa et al., 2004; van Erpecum et al., 2006; Sun et al., 2009; Cai and Chen, 2014; Wang Q. et al., 2017a; Wang G. et al., 2018a; Wang Y. et al., 2018b; Di Ciaula and Portincasa, 2018; Su et al., 2019; Wang et al., 2020; Wang et al., 2020; Wu et al., 2020; Zeng et al., 2021; Hu et al., 2022; Man et al., 2022). Current interventions for symptomatic GSD predominantly involve invasive surgical procedures, with a lack of prophylactic pharmaceutical options, underscoring the critical need for an enhanced understanding of GSD pathogenesis and the development of innovative treatment modalities (Ibrahim et al., 2018).

In recent years, long non-coding RNAs (lncRNAs) have emerged as pivotal regulators implicated in various diseases (Adams et al., 2017; Wang J. et al., 2017b; Li et al., 2019). As non-protein coding RNAs, lncRNAs govern diverse biological processes, including transcriptional and translational regulation, mRNA cleavage, and post-transcriptional modification, thereby exerting effects on epigenetic control, cell cycle regulation, differentiation, and immune response (Kurokawa, 2011; St Laurent et al., 2015; Kazimierczyk and Wrzesinski, 2021). The maturation of sequencing and gene chip technologies has facilitated the exploration of the intricate interactions between lncRNAs and their target protein-coding genes. This exploration has revealed complex regulatory networks that play a pivotal role in various life processes and disease progression in recent years. The association between lncRNA mutations or expression abnormalities and the onset and progression of numerous diseases has been documented. However, investigations into the role of lncRNAs in gallbladder stone formation are limited, and their specific functions in GSD remain elusive.

In this study, we used high-throughput sequencing to conduct expression profiling of lncRNAs and mRNAs in individuals with and without gallstones. Notably, this investigation represents the inaugural analysis of lncRNA expression in human gallbladder specimens with GSD. Our comprehensive analyses contribute to an exhaustive exploration, identifying potential candidate lncRNAs and their mRNA targets, thus laying the groundwork for further investigations into the mechanisms underlying gallstone formation.

## 2 Materials and methods

### 2.1 Participant and sample selection

All gallstone gallbladder tissue samples and normal gallbladder tissue samples (which were surgically removed due to liver hemangioma) were procured from the Second Affiliated Hospital of Kunming Medical University (located in Kunming, Yunnan province, China) between March 2021 and July 2021. The criteria employed for participant selection were delineated as follows: (i) individuals within the age range of 20–55 years, devoid of any history of antibiotic usage within the preceding 3 months; (ii) individuals without a medical history of viral hepatitis, malignant tumors, adenomyomatosis, chronic cholecystitis, underlying diseases, and metabolic disorders; (iii) verification of the absence of evident inflammation through pathological confirmation. Individuals meeting these criteria were randomly chosen for inclusion in the study. Participants in the control group were definitively diagnosed with liver IV-V segments, lacked any gallbladder lesions, but the gallbladder was inevitably removed due to the surgical approach, rendering it an ideal control group. Gallbladder tissue samples were procured from fresh surgical gallbladder specimens, frozen in liquid nitrogen, and subsequently stored at −80°C. High-throughput sequencing was used to analyze four paired samples, with an additional 10 paired samples collected for RT-qPCR validation analysis.

Approval for this study was granted by the ethics committee of The Second Affiliated Hospital of Kunming Medical University (Protocol Number: PJ-2021-79). The participants provided written informed consent to participate in this study. Additionally, written informed consent was obtained from the participants for the publication of any potentially identifiable images or data included in this article. Our research protocol adheres to the principles outlined in the Helsinki Declaration.

### 2.2 RNA extraction and library construction

RNA extraction, library construction, and sequencing procedures adhered to previously reported methodologies (Cui et al., 2022). Total RNA isolation and purification were conducted using TRIzol reagent (Invitrogen, Carlsbad, CA, USA) in accordance with the instructions of the manufacturer. The quantity and purity of each RNA sample were assessed using NanoDrop ND-1000 (NanoDrop, Wilmington, DE, USA). RNA integrity was assessed using Bioanalyzer 2,100 (Agilent, CA, USA) with a RIN number >7.0, and confirmation was obtained through electrophoresis with denaturing agarose gel. Approximately 2 μg of total RNA underwent ribosomal RNA removal as per the Epicentre Ribo-Zero Gold Kit manuscript (Illumina, San Diego, USA). Following purification, the ribo-minus RNA underwent fragmentation into small pieces using the Magnesium RNA Fragmentation Module (NEB, cat.e6150, USA) at 94°C for 5–7 min. Subsequently, the cleaved RNA fragments were reverse-transcribed to generate cDNA using SuperScript™ II Reverse Transcriptase (Invitrogen, cat. 1896649, USA). The cDNA was then employed to synthesize U-labeled second-stranded DNAs with *E. coli* DNA polymerase I (NEB, cat.m0209, USA), RNase H (NEB, cat.m0297, USA), and dUTP Solution (Thermo Fisher, cat.R0133, USA). An A-base was introduced to the blunt ends of each strand to facilitate ligation to indexed adapters. The above process is completed by the cooperation of T4 DNA polymerase, T4 polynucleotide kinase and Taq DNA polymerase. Each adapter featured a T-base overhang for ligating to the A-tailed fragmented DNA. Ligating single- or dual-index adapters to the fragments was followed by size selection using AMPureXP beads. After treatment with the heat-labile UDG enzyme (NEB, cat.m0280, USA) for the U-labeled second-stranded DNAs, the ligated products underwent PCR amplification under the following conditions: initial denaturation at 95°C for 3 min; eight cycles of denaturation at 98°C for 15 s, annealing at 60°C for 15 s, and extension at 72°C for 30 s; and a final extension at 72°C for 5 min. The average insert size for the final cDNA library was 300 ± 50 bp. Ultimately, 2 × 150 bp paired-end sequencing (PE150) was performed on an Illumina Novaseq™ 6000 (LC-Bio Technology CO., Ltd., Hangzhou, China) in accordance with the recommended protocol given by the vendor.

### 2.3 Data processing

The removal of reads containing adaptor contamination, low-quality bases, and undetermined bases was performed using fastp (Chen et al., 2018). Subsequent verification of sequence quality was conducted using fastp. Bowtie2 and Tophat2 were employed to map reads to the *Homo sapiens* GRCh38 genome (Langmead and Salzberg, 2012; Kim et al., 2013). The mapped reads for each sample underwent assembly using StringTie (Gupta et al., 2016). Subsequently, the transcriptomes from all samples were merged to reconstruct a comprehensive transcriptome, achieved through gffcompare (https://github.com/gpertea/gffcompare/). After generating the final transcriptome, StringTie was used to estimate the expression levels of all transcripts. Transcripts were annotated with known mRNAs, known lncRNAs, and transcripts shorter than 200 nt were discarded. CPC and CNCI were then used to predict transcripts with coding potential (Kong et al., 2007; Sun et al., 2013). Transcripts with CPC scores < −1 and CNCI scores <0 were removed. The remaining transcripts with class codes (I, j, o, u, x) were considered as lncRNAs. StringTie was used to determine the expression levels of mRNAs and lncRNAs by calculating Fragments Per Kilobase Million (FPKM = [total_exon_fragments/mapped_reads(millions) × exon_length(kB)]). Differentially expressed mRNAs (DEmRNAs) and lncRNAs (DElncRNAs) between gallstone and control samples were selected based on |log2 (fold change)| >1 and a *p*-value <0.05 using the “limma” R package. The location of DElncRNAs was visualized using the R package “circlize".

### 2.4 Functional enrichment of DElncRNAs and DEmRNAs

The investigation into the function of DElncRNAs involved the use of the Multi Experiment Matrix online tool (http://biit.cs.ut.ee/mem/index.cgi). This tool was used to predict the target genes of DElncRNAs by selecting the dataset “A-AFFY-44: Affymetrix GeneChip Human Genome U133 Plus 2.0 [HG-U133_PLUS_2]" encompassing 2,811 datasets. Subsequently, the “ClusterProfiler” R package was applied to conduct Gene Ontology (GO) and Kyoto Encyclopedia of Genes and Genomes (KEGG) pathway enrichment analyses for both DElncRNAs and differentially expressed messenger RNAs (DEmRNAs). Enriched GO and KEGG pathways were considered significant with an adjusted *p*-value threshold of <0.05. Additionally, Ingenuity Pathway Analysis (IPA) was employed to identify diseases and functions significantly associated with DElncRNAs and DEmRNAs, with a significance threshold set at *p*-value <0.05.

### 2.5 Construction of co-expression lnRNA-mRNA network and ceRNA network

To establish the co-expression network between DElncRNAs and DEmRNAs, Pearson’s correlations were computed. The criteria for co-expression were defined as |cor| ≥ 0.97 and *p*-value <0.0001. Subsequently, Cytoscape software was used for the construction and visualization of the co-expression network. For the prediction of miRNAs targeting DEmRNAs, the miRWalk, TargetScan, and miRDB databases were used. The starbase database was used to predict DElncRNAs interacting with miRNAs. Subsequently, miRNA-DEmRNA and miRNA-DElncRNA pairs were extracted based on their expression patterns to construct the ceRNA network.

### 2.6 Prediction of cis- and trans-regulation

For cis-regulation, the top five upregulated and top five downregulated DElncRNAs with the highest degrees in the co-expression network were selected. Following the definition of cis-regulation, we identified adjacent genes located within 300 kb upstream or downstream of the aforementioned 10 lncRNAs (Font-Cunill et al., 2018). Subsequently, these adjacent genes were intersected with DEmRNAs in the co-expression network. The resulting overlapped DEmRNAs were designated as cis-regulated genes. Regarding trans-regulation, the focus was on understanding how lncRNAs function through transcription factors (TFs). The TFcheckpoint database (http://tfcheckpoint.org) provided a list of 3,479 TFs, which was then intersected with DEmRNAs. Subsequently, the TRRUST database (https://www.grnpedia.org/trrust/) was used to predict the targets of the overlapped TFs. Finally, the lncRNA-TF-mRNA network was constructed and visualized using Cytoscape software.

### 2.7 RT-qPCR

Total RNA was extracted using Nuclezol LS RNA Isolation Reagent (ABP Biosciences Inc, China) following the instructions provided by the manufacturer. The concentration of RNA and the ratios of OD260/280 and OD260/230 were determined using a Nanophotometer N50 (OD260/280: 1.8-2.2, RNA concentrations ˃ 100 ng/ul). After determining the concentration and purity of the RNA, qualified samples underwent reverse transcription using the SureScript-First-strand-cDNA-synthesis-kit (GeneCopoeia, USA). Subsequently, qPCR was conducted on a CFX96 Real-time PCR System (Bio-Rad, USA) using BlazeTaq™ SYBR^®^ Green qPCR Mix 2.0 (GeneCopoeia, USA). The thermal cycling conditions comprised 40 cycles at 95°C for 30 s, 95°C for 10 s, 60°C for 20 s, and 72°C for 30 s. The 2^-△△Ct^ method was used to calculate gene expressions (Schmittgen and Livak, 2008).

### 2.8 Statistical analysis

All data are presented as means ± standard deviation. Comparisons between two groups were conducted using Student’s t-test. A *p*-value <0.05 was considered statistically significant.

## 3 Results

### 3.1 Identification and functional analysis of 304 DElncRNAs in gallstones

**FIGURE 1:**
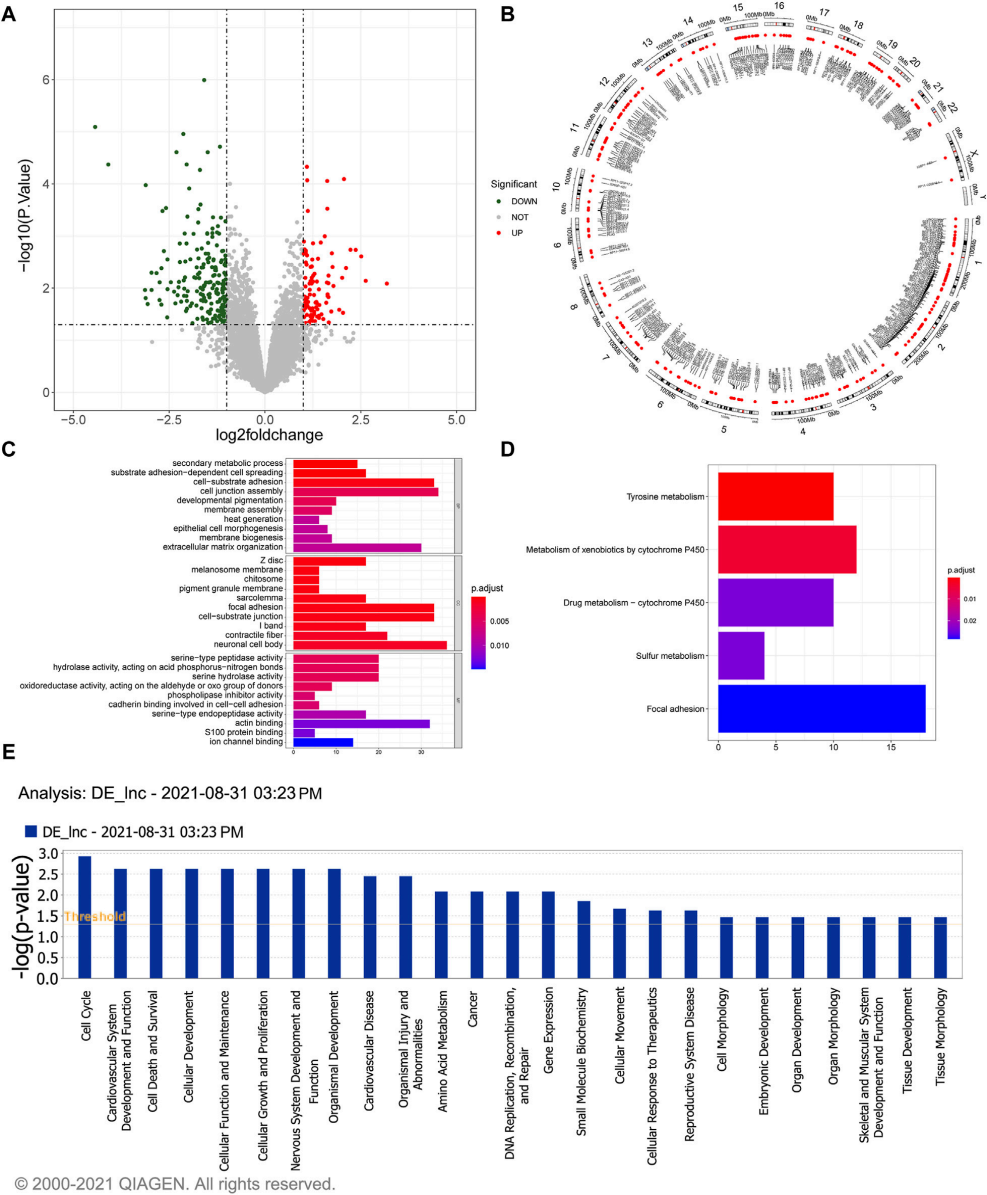
Identification and functional enrichment study of 304 DelncRNAs. **(A)** The volcano plot illustrates the differential expressed lncRNAs. The red dots represent significantly upregulated lncRNAs, the green dots represent significantly downregulated lncRNAs, and the gray dots represent lncRNAs without significant expression differences between the GSD and the control group. **(B)** The Circos image visually depicts the distribution of differentially expressed lncRNAs on chromosomes. **(C)** GO enrichment analysis of the differentially expressed lncRNAs. The horizontal axis reveals the gene count enriched in a particular GO entry, and the vertical axis indicates the GO terms. The color of the bars reflects the *p*-value, with redder colors indicating smaller *p*-values. **(D)** KEGG pathway enrichment analysis of differentially expressed lncRNAs. The horizontal axis represents the number of enrichments in the pathway. **(E)** Pathway analysis using IPA for differentially expressed lncRNAs.

A total of 304 DElncRNAs, comprising 103 upregulated and 201 downregulated lncRNAs in gallstone samples compared to control samples were identified (Figure 1A; Supplementary Table S1). The Circos image illustrated the widespread distribution of these DElncRNAs across all chromosomes (Figure 1B). A comprehensive list of 926 target genes was obtained (Supplementary Table S2), and they exhibited significant enrichment in 60 biological processes (BP), 37 cellular components (CC), and 23 molecular functions (MF) (Supplementary Table S3). The top 30 GO terms are depicted in Figure 1C, highlighting the involvement of potential target genes of DElncRNAs in various cellular functions. Furthermore, the potential target genes of DElncRNAs were notably enriched in KEGG pathways related to tyrosine metabolism, metabolism of xenobiotics by cytochrome P450, and drug metabolism-cytochrome P450 (Figure 1D). Additionally, Ingenuity Pathway Analysis (IPA) indicated that DElncRNAs may play roles in cell cycle regulation, cardiovascular system development and function, cell death and survival, and cellular development, among other processes (Figure 1E).

### 3.2 Identification and functional analysis of 934 DEmRNAs in gallstones

**FIGURE 2:**
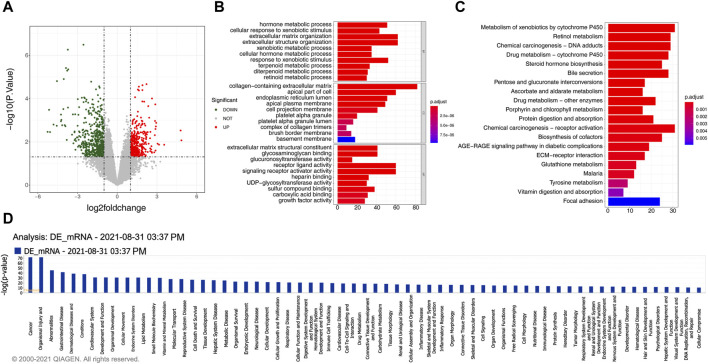
934 DEmRNAs selected for functional analysis. **(A)** The volcano plot displays the differential expression of mRNAs. The red dot indicates significantly upregulated mRNAs, the green dot represents significantly downregulated mRNAs, and the gray dot signifies mRNAs without significant expression differences between the GSD and the control group. **(B)** GO enrichment analysis of differentially expressed mRNAs. **(C)** KEGG pathway enrichment analysis of differentially expressed mRNAs. **(D)** Pathway analysis using IPA for differentially expressed mRNAs.

A total of 934 DEmRNAs, comprising of 396 upregulated and 538 downregulated mRNAs in gallstone samples were identified (Figure 2A, Supplementary Table S4). The DEmRNAs exhibited significant enrichment in 723 biological processes (BPs), 48 CCs, 96 MFs, and 26 KEGG pathways (Supplementary Table S5, Supplementary Table S6). These DEmRNAs were primarily associated with metabolic processes and extracellular matrix organization, including hormone metabolic processes, terpenoid metabolic processes, and diterpenoid metabolic processes (Figure 2B). Consistent with the GO enrichment results, the DEmRNAs were predominantly enriched in metabolism and extracellular matrix-related pathways, such as retinol metabolism, porphyrin and chlorophyll metabolism, and ECM-receptor interaction (Figure 2C). Moreover, the DEmRNAs were closely related to the metabolic effects of cytochrome P450 on exogenous substances, biosynthesis of steroid hormones, bile secretion, metabolism and protein digestion and absorption, biosynthesis of cofactors, and pentose and glucuronic acid conversion metabolic pathways, among others (Figure 2D).

### 3.3 Cis- and trans-regulatory functions of DElncRNAs in GSD

**FIGURE 3:**
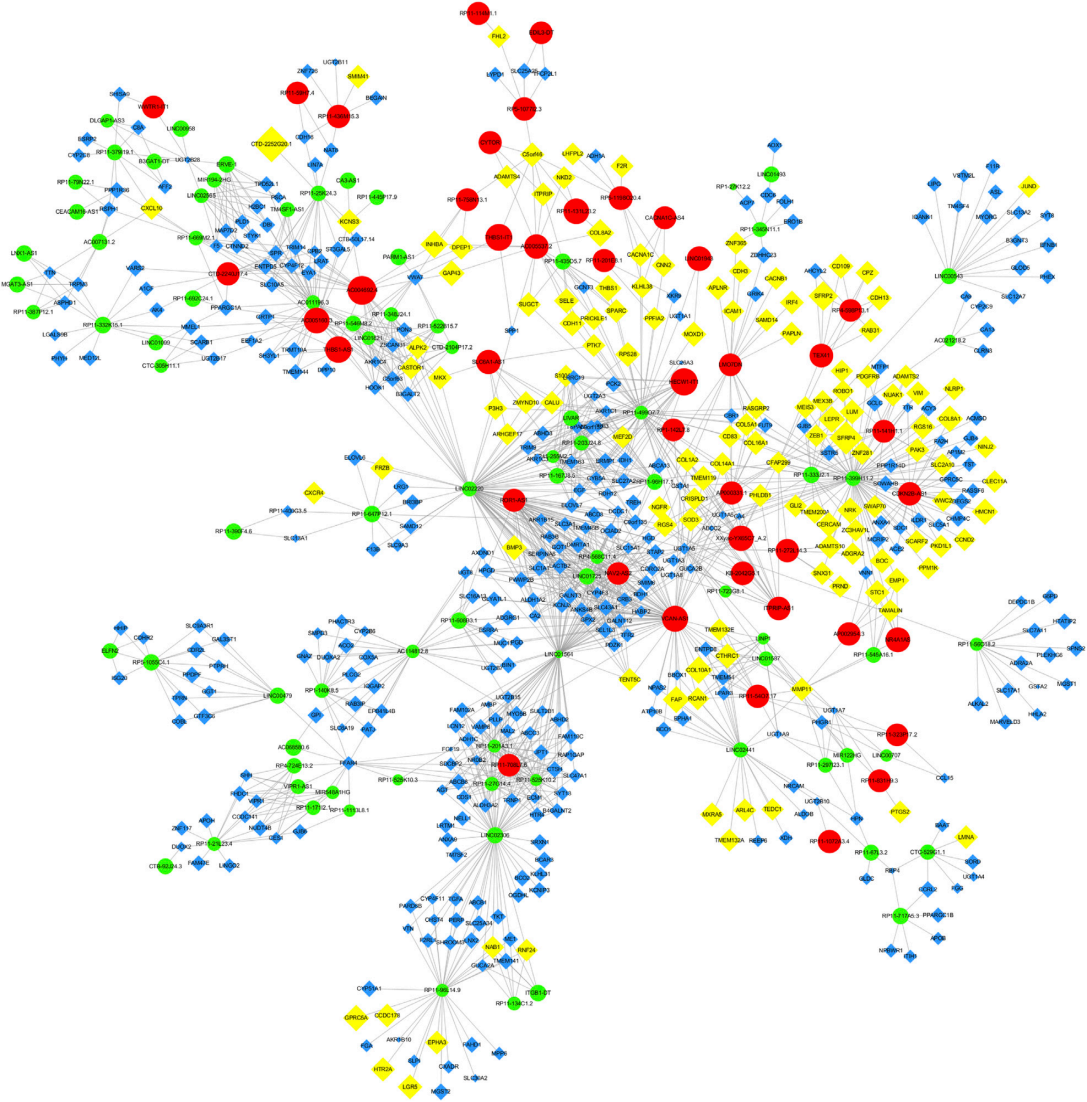
Co-expression network between DElncRNAs and DEmRNAs.

**FIGURE 4:**
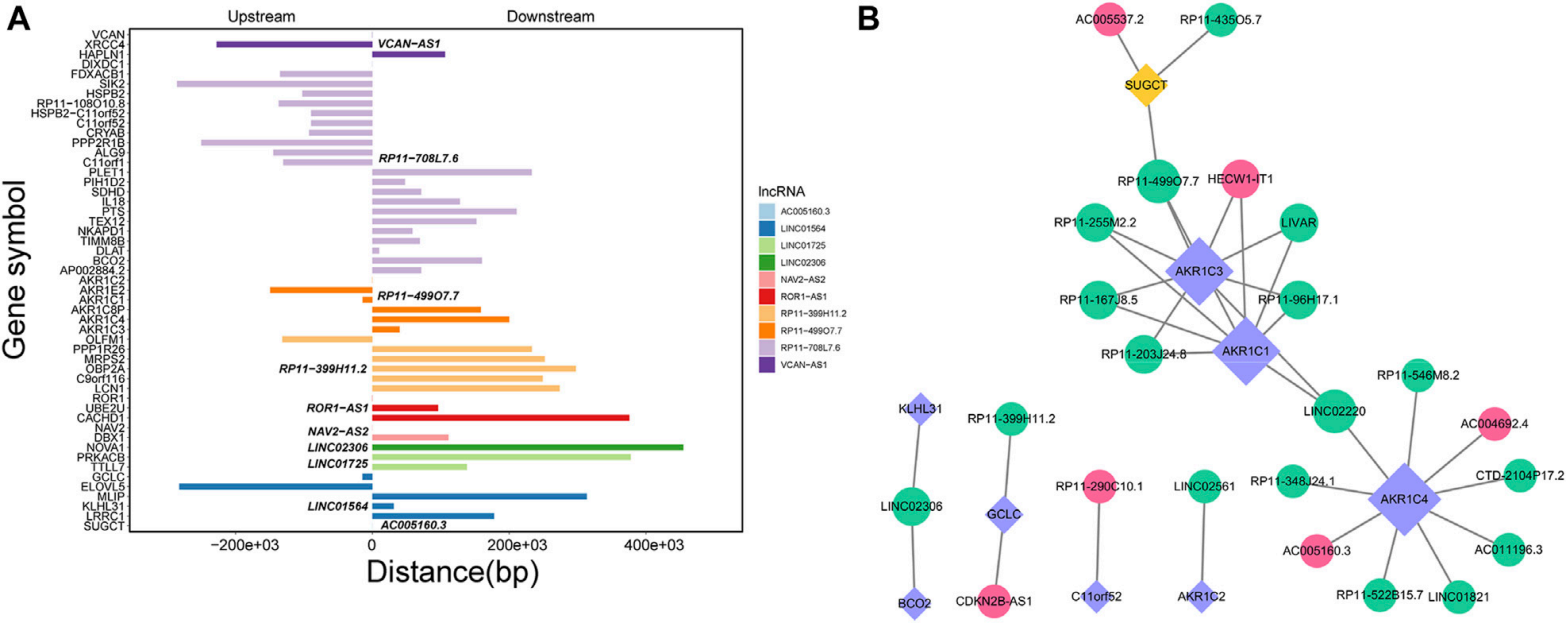
**(A)** Analysis of cis-regulation involving 10 DElncRNAs and their adjacent mRNAs. **(B)** Network illustrating the cis-regulation interactions between the 10 DElncRNAs and their adjacent DEmRNAs.

Through Pearson’s correlation analysis, 597 DEmRNAs were found to be strongly correlated with 194 DElncRNAs. Subsequently, a co-expression network was constructed and visualized using Cytoscape software (Figure 3). After excluding lncRNAs with no neighboring mRNAs, the top 10 lncRNAs (5 upregulated and five downregulated) with correlations in the lncRNA-mRNA network were considered as target lncRNAs. These included RP11-399H11.2, VCAN-AS1, LINC01564, RP11-499O7.7, LINC02306, ROR1-AS1, NAV2-AS2, LINC01725, AC005160.3, and RP11-708L7.6. A total of 51 mRNAs were found to be cis-regulated with 10 target lncRNAs, and the number of adjacent mRNAs for each lncRNA varied (e.g., RP11-399H11.2 had 21 adjacent mRNAs, while VCAN-AS1 had only two adjacent mRNAs) (Figure 4A). After overlapping adjacent mRNAs with mRNAs in the co-expression network, a cis-regulation network composed of 32 nodes and 34 edges (Figure 4B) was constructed using Cytoscape software. For example, the downregulated BCO2 and KLHL31 were cis-regulated by LINC02306.

**FIGURE 5:**
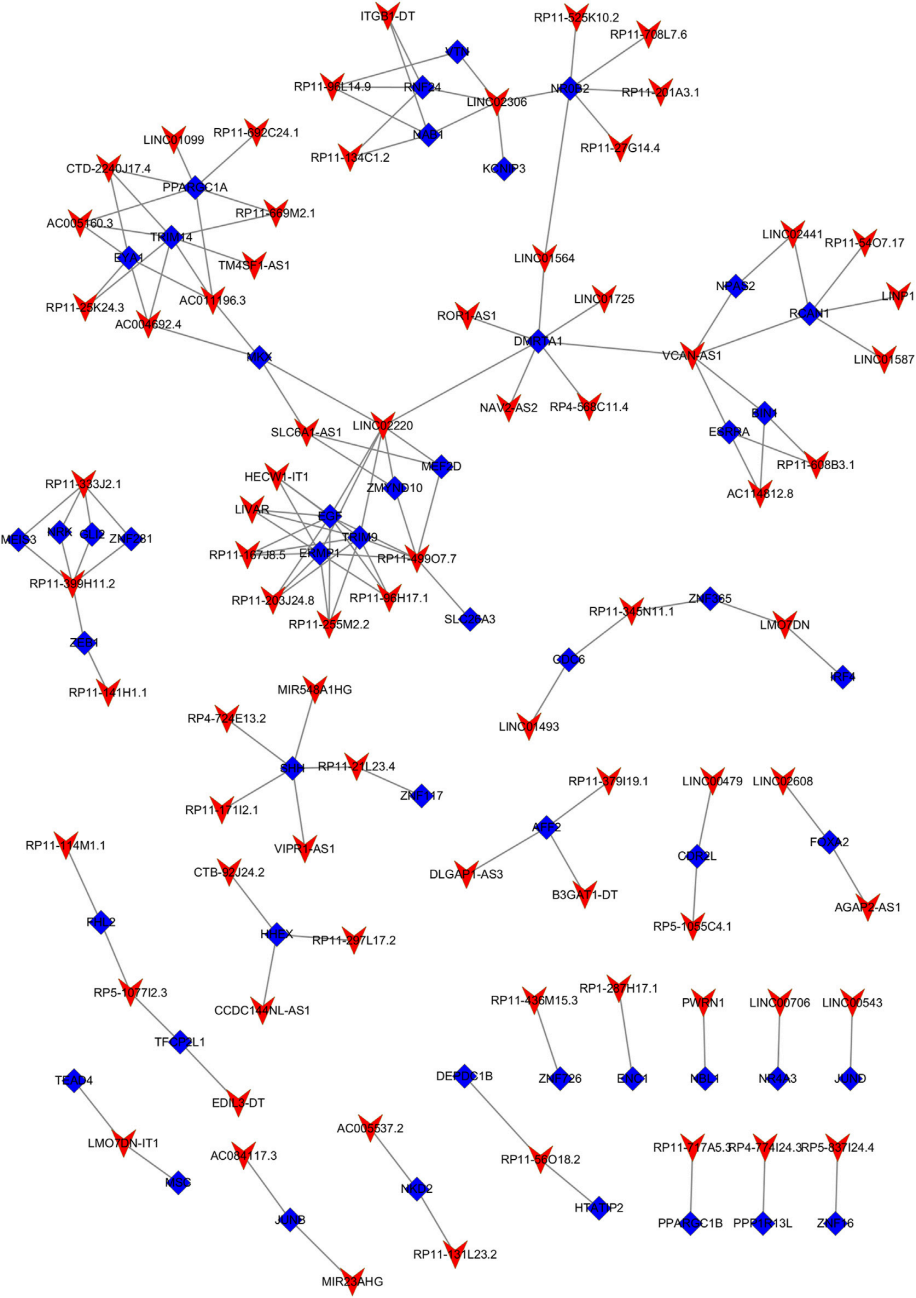
TFs (DEmRNAs)-lncRNAs regulatory network.

**FIGURE 6:**
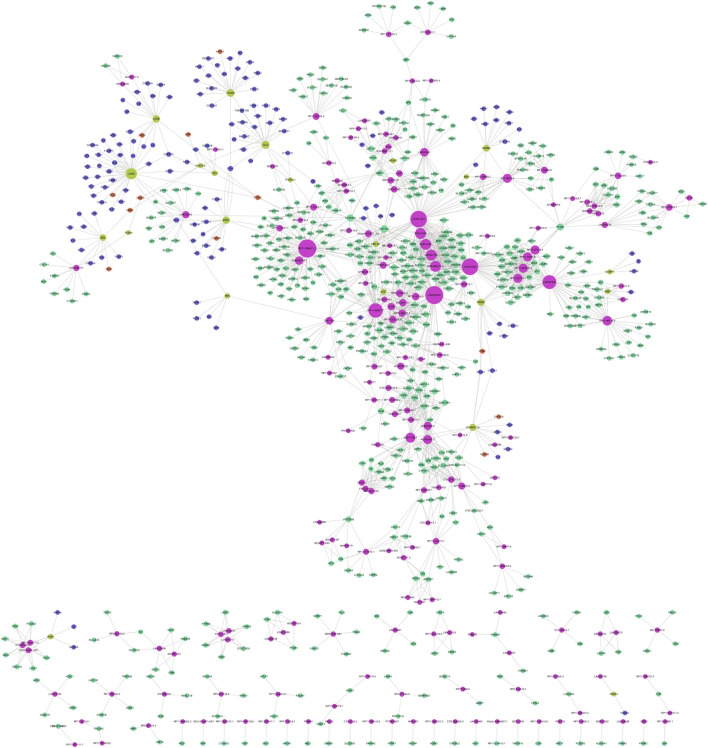
lncRNA-TF-mRNA regulatory network.

Simultaneously, the trans-regulatory function of lncRNAs was investigated. Initially, 50 TFs were obtained by overlapping DEmRNAs with TFs in the Tfcheckpoint database. The corresponding TFs (DEmRNAs)-lncRNAs pairs were then extracted from the lncRNA-mRNA co-expression network (Figure 5). Subsequently, using the TRRUST database, 22 TFs out of 55 were predicted to regulate the transcription of 150 targets, with 13 targets being in the co-expression network. Finally, using Cytoscape software, the TF-mRNA and lncRNA-mRNA co-expression networks were integrated to construct the lncRNA-mRNA-TF regulatory network, comprising 927 nodes and 1,927 edges (Figure 6).

### 3.4 Construction of ceRNA network in gallstones

**FIGURE 7:**
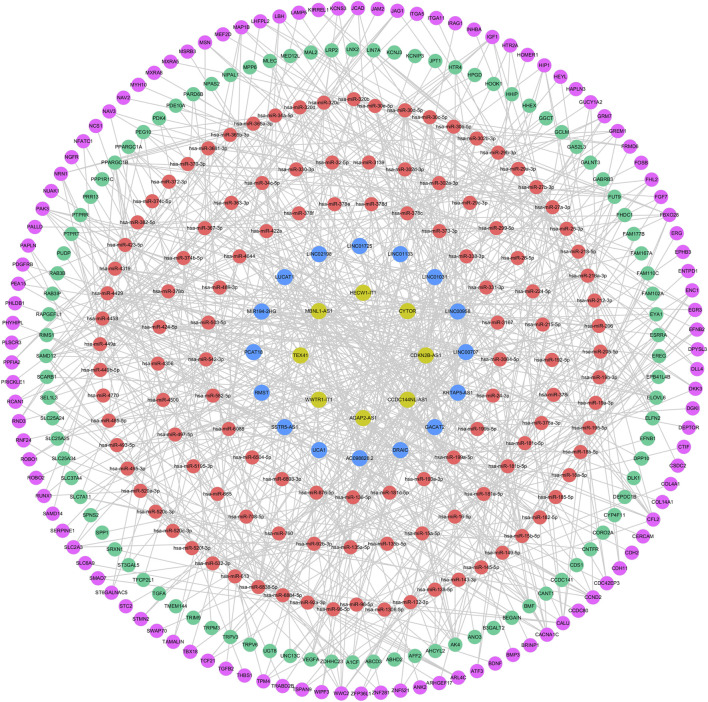
ceRNA regulatory network. The pink circle represents upregulated mRNAs, the green circle represents downregulated mRNAs, the red circle represents miRNAs, the yellow circle represents upregulated lncRNAs, and the blue circle represents downregulated lncRNAs.

A total of 245 miRNAs were identified to target 289 DEmRNAs based on information from the miRWalk, TargetScan, and miRDB databases. Additionally, 220 miRNAs interacting with 24 DElncRNAs were predicted through the starbase database. Considering the expression patterns of mRNA-miRNA and miRNA-lncRNA pairs, a comprehensive network was constructed, consisting of 24 DElncRNAs, 201 DEmRNAs, and 120 predicted miRNAs, to form the ceRNA network (Figure 7). The 24 DElncRNAs involved in the ceRNA network encompassed eight upregulated DElncRNAs and 16 downregulated DElncRNAs.

### 3.5 Verification of DEmRNAs and DElncRNAs by RT-qPCR

**FIGURE 8:**
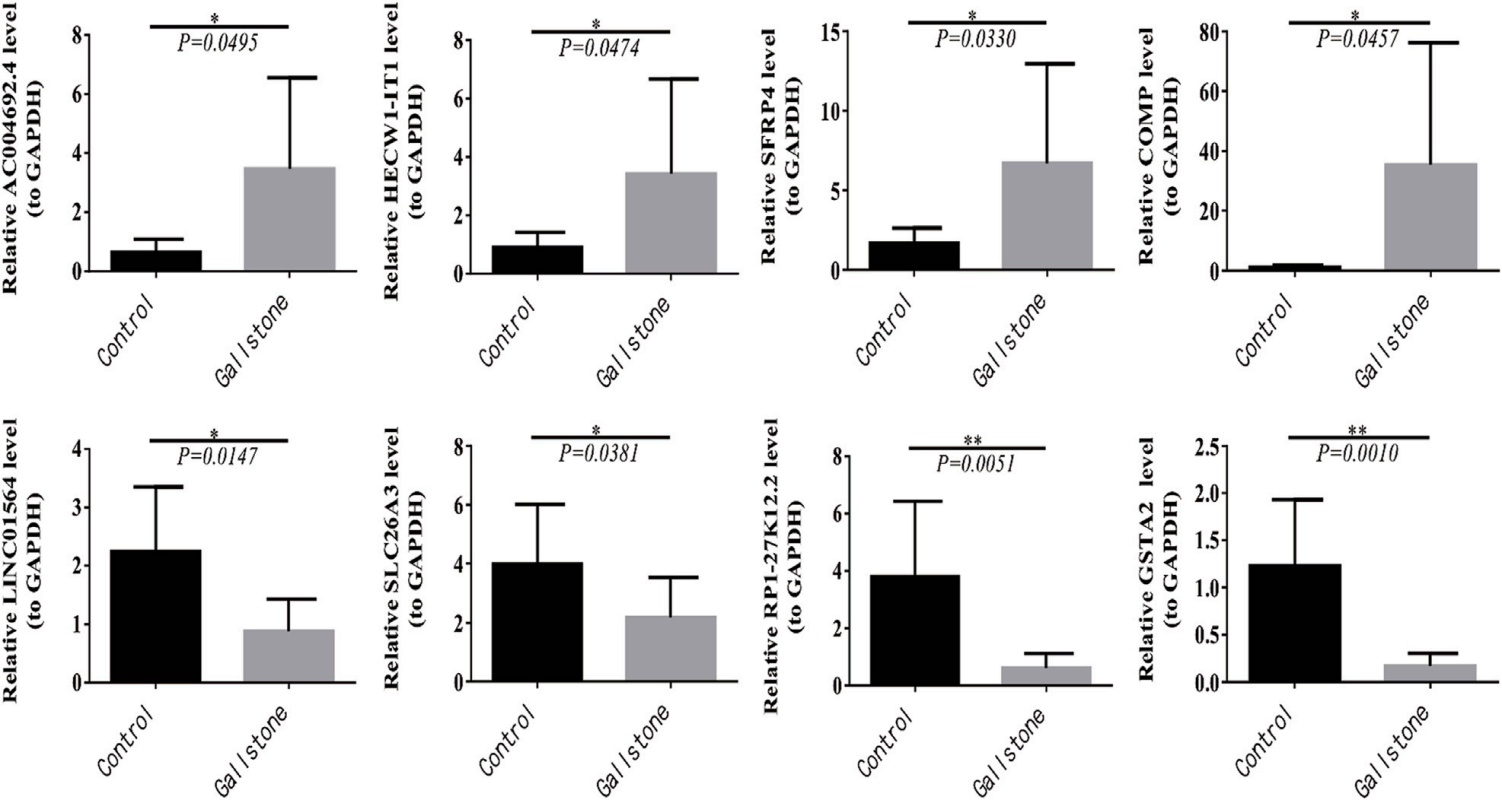
Four lncRNAs and four mRNAs were validated for expression in control and GSD samples with RT-qPCR.

Furthermore, we conducted RT-qPCR to validate the findings obtained from the sequencing results. The top two upregulated and downregulated DEmRNAs and DElncRNAs between gallstone and control samples were selected for validation. The top two upregulated lncRNAs were AC004692.4 and HECW1-IT1, while the top two downregulated lncRNAs were RP1-27K12.2 and LINC01564. Similarly, the top two upregulated mRNAs were SFRP4 and COMP, and the top two downregulated DEmRNAs were SLC26A3 and GSTA2. A *t*-test was used to compare the expression differences of these four lncRNAs and four mRNAs between gallstone and control samples. The results indicated that AC004692.4, HECW1-IT1, SFRP4, and COMP were significantly higher expressed in gallstone samples, whereas LINC01564, SLC26A3, RP1-27K12.2, and GSTA2 were markedly lower expressed in gallstone samples compared to control samples (Figure 8). These findings were consistent with the results obtained from the sequencing, reinforcing the reliability of the sequencing data.

## 4 Discussion

In this study, we investigated the differential expression of lncRNAs and mRNAs between patients with GSD and controls using sequencing technology. Functional enrichment analysis of these differentially expressed genes revealed associations with cytochrome P450, biosynthesis of steroid hormones, bile secretion, and other processes closely related to gallstone formation.

While some researchers have conducted transcriptome sequencing on gallbladder polyps and gallbladder stones in gallbladder epithelial tissue, and others have constructed animal models of gallbladder stones in mice using healthy mice as controls for sequencing and bioinformatics analysis, these approaches have limitations (Yang et al., 2015). Due to the similarity in the causes and mechanisms of gallbladder cholesterol polyps and gallbladder stones, and the inability of animal models to fully replicate the human disease state, they may not serve as the most ideal control groups. In this project, the control group included patients undergoing surgical treatment for hepatic hemangiomas of liver segments IV-V, where gallbladder tissue could not be preserved during surgery. Preoperative examinations indicated normal gallbladder size, morphology, thin and smooth walls, and clear bile. Postoperative anatomical specimens revealed no sediment deposition in bile, clear bile, and pathological examination confirmed normal gallbladder tissue. Therefore, the control group sample selected for this project may closely resemble the state of a normal gallbladder in a healthy human body, making it more comparable.

In cellular contexts, cytochrome P450 is predominantly distributed on the endoplasmic reticulum and intramitochondrial membrane, serving as a terminal oxygenase involved in the synthesis of sterol hormones and other processes. Cytochrome P450 genes, such as CYP7A1, CYP7B1, CYP27A1, catalyze various steps in cholesterol catabolism and bile acid synthesis (Lee et al., 2018). As a regulator of cholesterol, steroid biosynthesis can influence gallbladder contraction and alter the expression of factors regulating cholesterol metabolism, promoting the secretion of lithogenic bile and the formation of stones (Reshetnyak, 2012). These identified biological processes and enriched pathways, including protein transport and lipid metabolism, are directly or indirectly implicated in the regulation of gallbladder stone pathogenesis, aligning with our anticipated results.

Subsequently, we used RT-qPCR to validate the expression levels of the top two upregulated and downregulated DEmRNAs and DElncRNAs, respectively. The results of the detection were in line with the sequencing outcomes, indicating the accuracy and reliability of the sequencing results. Notably, considering that the expression of these eight DEmRNAs and DElncRNAs exhibited the most significant differences between the two groups, we hypothesize that they may play pivotal roles in the initiation and progression of gallbladder stones. Among these candidates, secreted frizzled-related protein 4 (SFRP4) stands out as the largest member of the SFRP family and has been strongly associated with various diseases, including obesity, type 2 diabetes, and malignancy (Bukhari et al., 2019). Research has linked human SFRP4 expression to insulin sensitivity, triglyceride levels, and the promotion of *de novo* synthesis pathway and glycolysis in liver fat, leading to impaired islet β cell function, heightened lipid accumulation, and insulin resistance in the liver (Brix et al., 2016; Bukhari et al., 2019; Hörbelt et al., 2019; Zhang et al., 2020). Accumulated lipids and insulin resistance can result in increased hepatic cholesterol secretion, reduced bile acid secretion, and gallbladder dyskinesias, fostering the development of supersaturated bile and potentially promoting gallstone formation (Tsai et al., 2008). The significant upward trend of SFRP4 in the stone group observed in this study provides further support for these findings, suggesting the potential of SFRP4 as a biomarker for gallbladder stones and its crucial role in regulating human cholesterol balance.

Glutathione S-transferases (GSTs) are crucial liver detoxification enzymes with significant roles in bile acid metabolism (Xie et al., 2005). Within the cytoplasmic GSTs family, Glutathione S-transferase alpha (GSTA) superfamily plays a pivotal role in metabolizing endogenous toxic compounds. GSTA can catalyze a wide range of substrates, including bile acids, reactive oxygen species produced under cholestasis, and non-polar compounds. Previous studies have noted a substantial reduction in GSTA1-4 in human obstructive cholestasis due to gallstone biliary obstruction (Chai et al., 2015). In the context of gallstone formation, characterized by cholestasis, our findings reveal a notable downregulation of GSTA2 in the gallstone group, indicating a weakened detoxification capacity of the liver against toxic bile acids. The specific downregulation mechanism of GSTA2 in this process warrants further investigation.

Furthermore, the downregulation of LINC01564 has been associated with genomic instability and metabolic adaptation in liver cancer, while SLC26A3 has been linked to inflammatory bowel disease and infectious diarrhea (Yu, 2021; Zhang et al., 2021; Chen et al., 2022). The upregulation of COMP and HECW1-IT1 is more commonly reported in cancer, and their regulatory roles in the mechanism of gallstone formation require further exploration. The remaining genes, not yet reported, await future discoveries to elucidate their potential roles.

In addition to elucidating the pathological mechanisms underlying the development of gallstones, it is crucial to further explore the clinical implications of these differentially expressed genes. Investigating whether these genes can serve as serological markers for predicting cholelithiasis, their potential correlation with disease severity, and the efficacy of combined gene detection in enhancing diagnostic accuracy are essential areas of inquiry. Furthermore, assessing the feasibility of utilizing these genes as therapeutic targets to mitigate disease progression and potentially avoid surgical intervention is of utmost importance. Additionally, exploring their utility as indicators for detecting recurrence post-cholecystolithotomy and as targets for preventive strategies warrants thorough investigation. Future endeavors will involve expanding the sample size and conducting multicenter studies to establish a robust clinical foundation for utilizing these differential genes as early diagnostic markers or therapeutic targets for gallstone management, complemented by animal experimentation to validate these findings.

## 5 Conclusion

In conclusion, this study provides a comprehensive insight into the expression profiles of lncRNAs and mRNAs in GSD, shedding light on potential mechanisms underlying gallstone formation. The findings offer a novel perspective for investigating the molecular mechanisms involved in the pathogenesis and progression of gallbladder stones. While SFRP4 and GSTA2 are identified as potentially significant regulators in GSD, the precise molecular regulatory mechanisms require further investigation, pointing towards future research directions.

## Data Availability

The datasets presented in this study can be found in online repositories. The names of the repository/repositories and accession number(s) can be found in the article/Supplementary Material.

## Supplementary material

SUPPLEMENTARY TABLE S1 Patient information.

SUPPLEMENTARY TABLE S2 304 DElncRNAs in gallstone samples.

SUPPLEMENTARY TABLE S3 926 target genes were predicted by DE-lncRNA.

SUPPLEMENTARY TABLE S4 GO analysis results of 926 target genes.

SUPPLEMENTARY TABLE S5 934 DEmRNAs in gallstone samples.

SUPPLEMENTARY TABLE S6 GO analysis results of DEmRNAs.

SUPPLEMENTARY TABLE S7 KEGG pathways results of DEmRNAs.

SUPPLEMENTARY FIGURE S1 RT-qPCR dissolution and amplification curves of AC004692.4, HECW1-IT1, RP1-27K12.2, LINC01564.

SUPPLEMENTARY FIGURE S2 RT-qPCR dissolution and amplification curves of SFRP4, COMP, SLC26A3, and GSTA2.

## Abbreviations

Long non-coding RNAs, (lncRNAs); messenger RNAs, (mRNAs); RiboNucleic Acid, (RNA); competing endogenouse RNA, (ceRNA); Quantitative Real-time Polymerase Chain Reaction, (RT-qPCR); Transcriptionfactor, (TF); Gene ontology, (GO); Kyoto Encyclopedia of Genes and Genomes, (KEGG); biological processes, (BP); cellular components, (CC); molecular functions, (MF); cytochrome P450 family 7 subfamily members 1, (CYP7A1); Ingenuity Pathway Analysis, (IPA); secreted frizzled-related protein 4, (SFRP4); Glutathione S-transferase, (GST); Glutathione S-transferase alpha, (GSTA).
