# Supplementary figures and images for: Comprehensive analysis of differential long non-coding RNA and messenger RNA expression in cholelithiasis using high-throughput sequencing and bioinformatics
Source: Front Genet. 2024 May 14;15:1375019. doi: 10.3389/fgene.2024.1375019 (PMC11130440; doi:10.3389/fgene.2024.1375019)

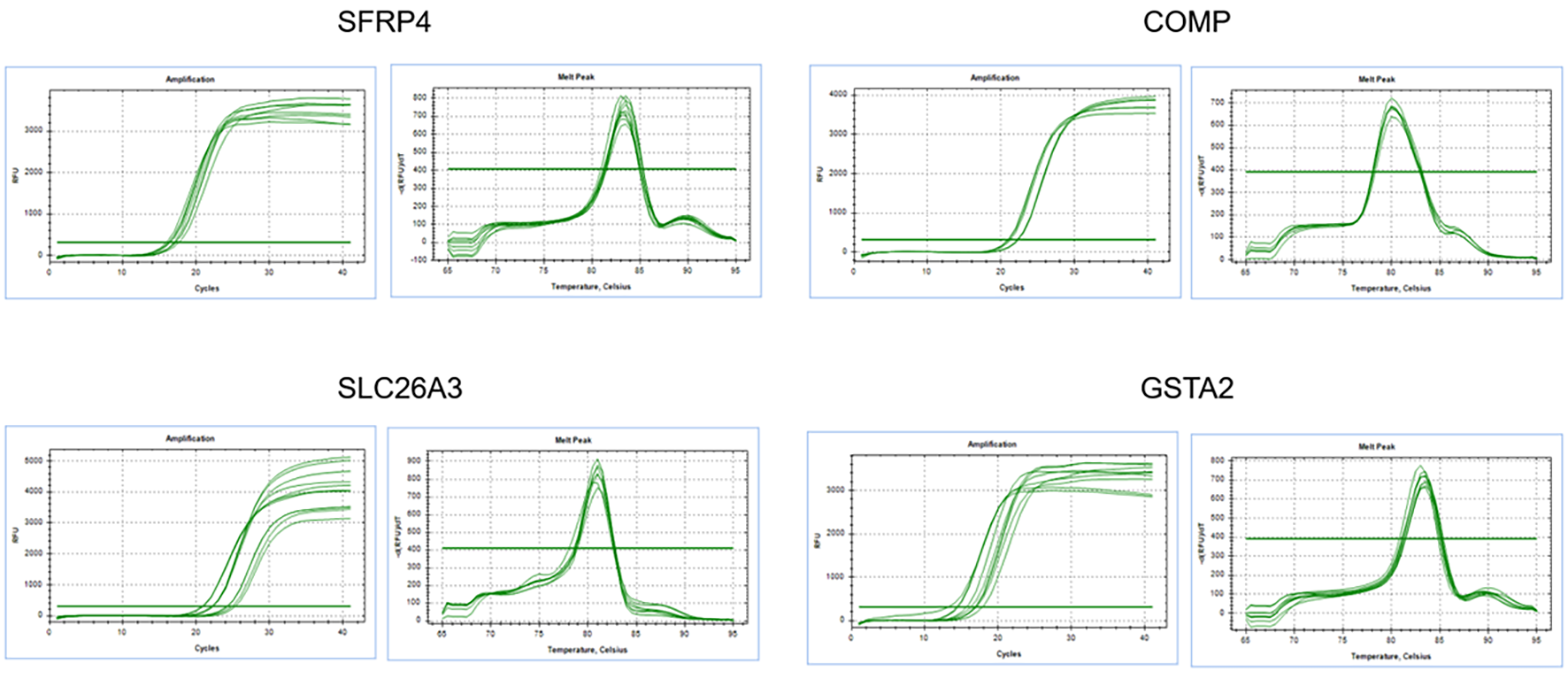

Supplement: Supplementary file 3 [file Image2.TIF]

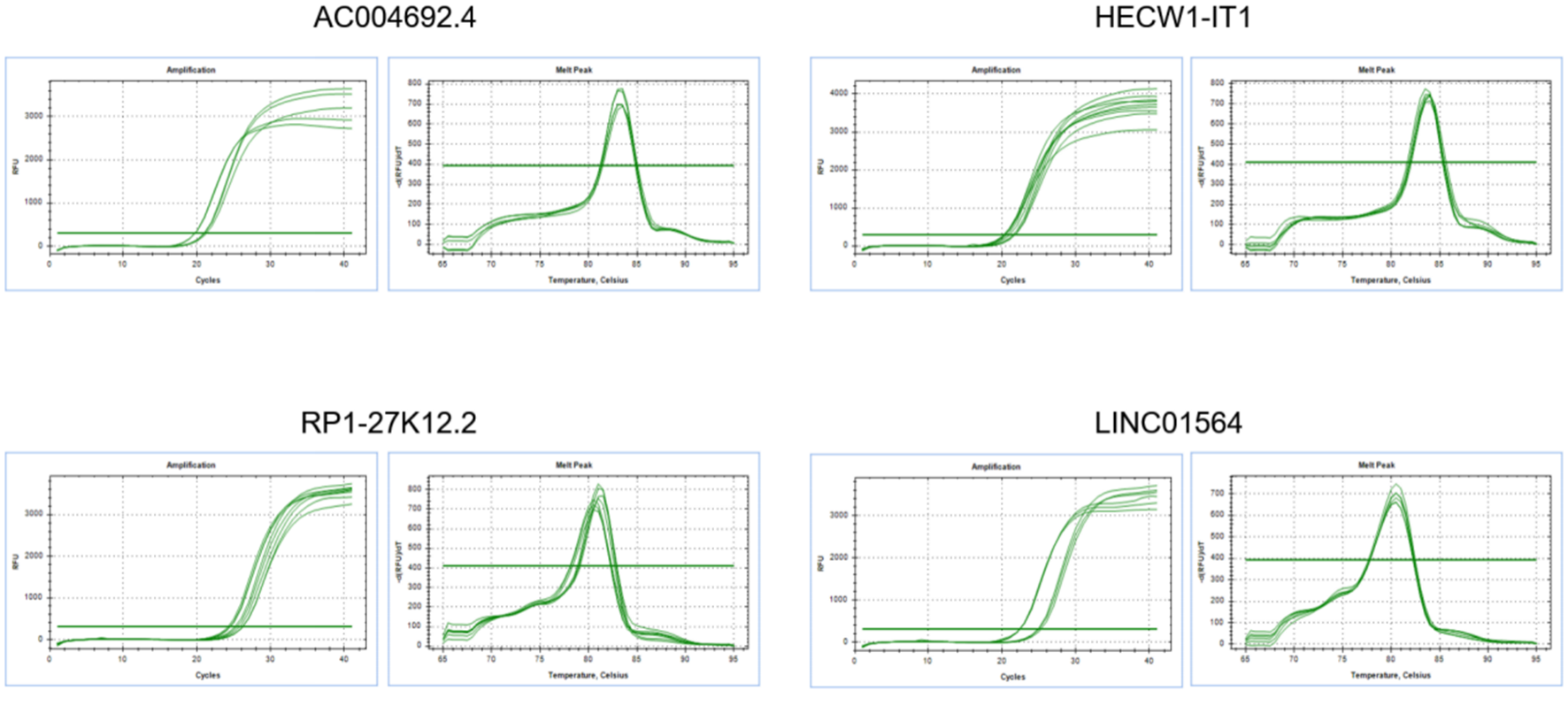

Supplement: Supplementary file 4 [file Image1.TIF]
